# Suspected fibrosing cholestatic hepatitis in an immunocompetent child with hepatitis C: Case report

**DOI:** 10.1002/jpr3.70250

**Published:** 2026-10-15

**Authors:** Sara Bohon, Regino Gonzalez‐Peralta, Robert J. Fontana, Richard Cordle, Robin LeGallo, Frank DiPaola

**Affiliations:** ^1^ Division of Pediatric Gastroenterology, Hepatology, and Nutrition University of Virginia Health System Charlottesville Virginia USA; ^2^ AdventHealth for Children AdventHealth Transplant Institute Orlando Florida USA; ^3^ Division of Gastroenterology & Hepatology University of Michigan Medical School Ann Arbor Michigan USA; ^4^ Division of Pediatric Gastroenterology and Nutrition Virginia Tech Carilion School of Medicine Roanoke Virginia USA; ^5^ Department of Pathology University of Virginia Health System Charlottesville Virginia USA

**Keywords:** direct‐acting antiviral therapy, hyperbilirubinemia, liver, pediatric

## Abstract

A 12‐year‐old boy presented to our center with elevated aminotransferases, jaundice, right upper quadrant tenderness, and hepatomegaly. Evaluation revealed a highly elevated blood hepatitis C virus (HCV) ribonucleic acid (RNA) level, and liver biopsy showed canalicular and intrahepatic cholestasis, feathery degeneration of hepatocytes, and periportal/perisinusoidal fibrosis. Though there were atypical biopsy features, including absence of bile ductular reaction, we diagnosed suspected fibrosing cholestatic hepatitis (FCH). FCH secondary to HCV infection has been reported exclusively in immunosuppressed adults. To our knowledge, this is the first reported case of suspected FCH in a child, and the first reported case of suspected HCV‐related FCH in any immunocompetent patient. Our patient was successfully treated with sofosbuvir/velpatasvir with sustained virologic response at 12 weeks. We conclude that FCH should be considered on the differential whenever any child with HCV infection presents with evidence of worsening liver injury including new hepatomegaly or jaundice.

## 1 INTRODUCTION

Fibrosing cholestatic hepatitis (FCH) is a severe and rapidly progressive form of liver injury first described in 1991 in adults following liver transplantation for chronic hepatitis B virus (HBV) infection.^1^

FCH has been nearly exclusively described in adults with chronic HBV or hepatitis C virus (HCV) who are immunosuppressed either following solid organ transplantation, related to human immunodeficiency virus (HIV), or as a consequence of treatment for autoimmune disease or malignancy. There has been only one reported case of HBV‐related FCH in an apparently immunocompetent adult^2^ and, to our knowledge, there have been no cases of FCH reported in children with or without immunocompromise. Here, we describe the development of suspected FCH in an apparently immunocompetent child with HCV genotype 1 infection.

## 2 CASE REPORT

A 12‐year‐old Caucasian male patient presented to an outside hospital for evaluation of a several‐month history of nausea and fatigue, and a 4‐week history of jaundice and pruritus with right upper quadrant tenderness and hepatomegaly. His body mass index (BMI) at presentation to our center was 17.05 kg/m^2^ (28th percentile), and he had no features of metabolic syndrome. Laboratory evaluation at presentation showed platelet count 502 × 10^3^/µL, albumin 4.8 g/dL, alanine transaminase (ALT) 703 U/L, aspartate transaminase (AST) 1631 U/L, total bilirubin 7.2 mg/dL, direct bilirubin 6.0 mg/dL, Alk Phos 310 U/L, gamma‐glutamyl transferase (GGT) 194 U/L, and INR 1.0.

Further diagnostic testing was remarkable for a blood hepatitis C viral ribonucleic acid (RNA) level as high as 7.24 log IU/mL, HCV genotype 1a. Magnetic resonance imaging of the abdomen confirmed significant hepatomegaly without liver nodularity or focal liver lesions, liver vascular disease, gallbladder or biliary disease, splenomegaly or other stigmata of portal hypertension. Testing for other viruses including HAV (anti‐HAV immunoglobulin M), HBV (HBsAg and total anti‐HBc), parvovirus (plasma polymerase chain reaction [PCR]), acute epstein‐barr virus and cytomegalovirus (serologies), and HIV (antigen/antibody and PCR) was negative. Testing for autoimmune liver disease (antinuclear antibody, anti‐actin, anti‐liver‐kidney microsomal, antimitochondrial antibody negative, and immunoglobulin G) was negative/normal, and alpha‐1 antitrypsin mutation analysis and Wilson disease testing (ceruloplasmin, 24‐h urine copper quantification, eye exam for Kayser Fleischer rings) were negative.

Past medical history was significant only for attention deficit hyperactivity disorder. He had been treated with atomoxetine, but this was stopped 6 months before presentation. He was not taking any other prescription or over‐the‐counter medications or herbal/dietary supplements. He had no history of recurrent or severe bacterial, viral, or fungal infections. Serum acetaminophen level and urine drug screen were negative. There was no known family history of liver disease including HCV, although his mother had a reported history of intravenous drug use. There was no known family history of immunodeficiency or autoimmune disorders. The patient had no history of alcohol use, Intravenous (IV) drug use, blood transfusions, tattoos or international travel.

The patient had persistent cholestasis for 4 weeks after his presentation and was referred to our center, whereupon he was admitted for percutaneous liver biopsy (Figure 1). The biopsy showed mild expansion of all portal tracts with a mixed inflammatory infiltrate, predominantly lymphocytes with scattered eosinophils and neutrophils and few plasma cells, focal interface activity, and a mild mixed lobular inflammatory infiltrate. There was no bile ductular proliferation. The lobules showed canalicular and intrahepatic cholestasis with feathery degeneration of hepatocytes (these changes were most pronounced in the periportal area), and hepatocyte swelling. Trichrome stain showed periportal and perisinusoidal fibrosis and focal early bridging fibrosis. The overall clinicopathologic findings were considered suggestive of HCV‐associated FCH. The liver biopsy slides were sent to an experienced hepatopathologist at an outside pediatric center for second opinion, and the team concurred with our assessment. We did consider the possibility of atomoxetine‐induced liver injury, however this was felt to be unlikely on clinical grounds including the timing of atomoxetine discontinuation. The Prevention Genetics Cholestasis Panel was negative (see Table S1 for list of genes included).

**Figure 1:**
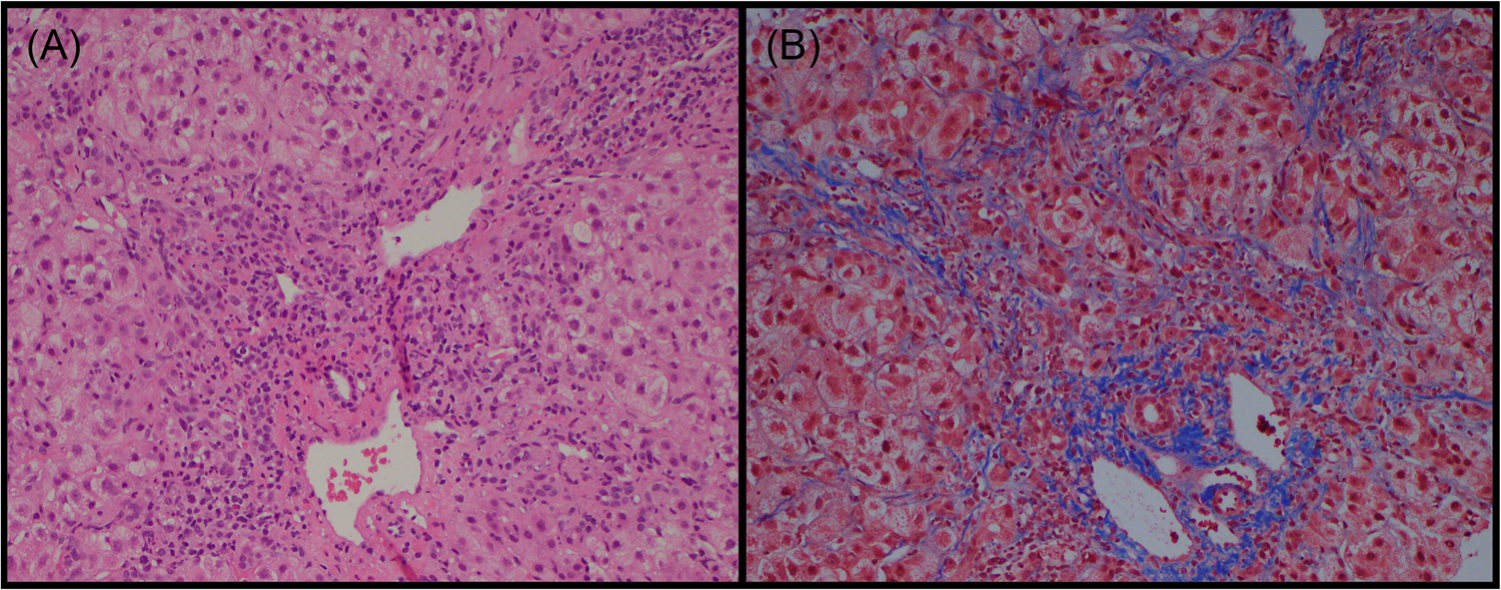
Liver biopsy findings. (A) H&E stain. The portal tract is mildly expanded with a mixed inflammatory infiltrate (predominantly lymphocytes with eosinophils and neutrophils) and minimal interface activity. The periportal hepatocytes show feathery degeneration. (B) Trichrome stain. There is prominent periportal and perisinusoidal fibrosis. H&E, hematoxylin and eosin.

He was prescribed a 12‐week course of sofosbuvir‐velpatasvir, and then reevaluated at 40 days into the treatment course. At that time, he was noted to have persistent but significantly improved hepatomegaly, and dramatically improved laboratories including ALT 40 U/L, AST 39 U/L, total bilirubin 0.3 mg/dL, Alk Phos 147 U/L, and GGT 55 U/L, and nearly undetectable blood HCV RNA PCR at <1 log IU/mL. At 12 weeks post completion of treatment, pruritus and hepatomegaly had completely resolved, laboratory tests had normalized, and HCV RNA PCR was negative (Figure 2). Therefore, the patient achieved SVR12, defined as undetectable HCV RNA 12 weeks after completion of therapy. No adverse effects of therapy or unanticipated events were noted.

**Figure 2:**
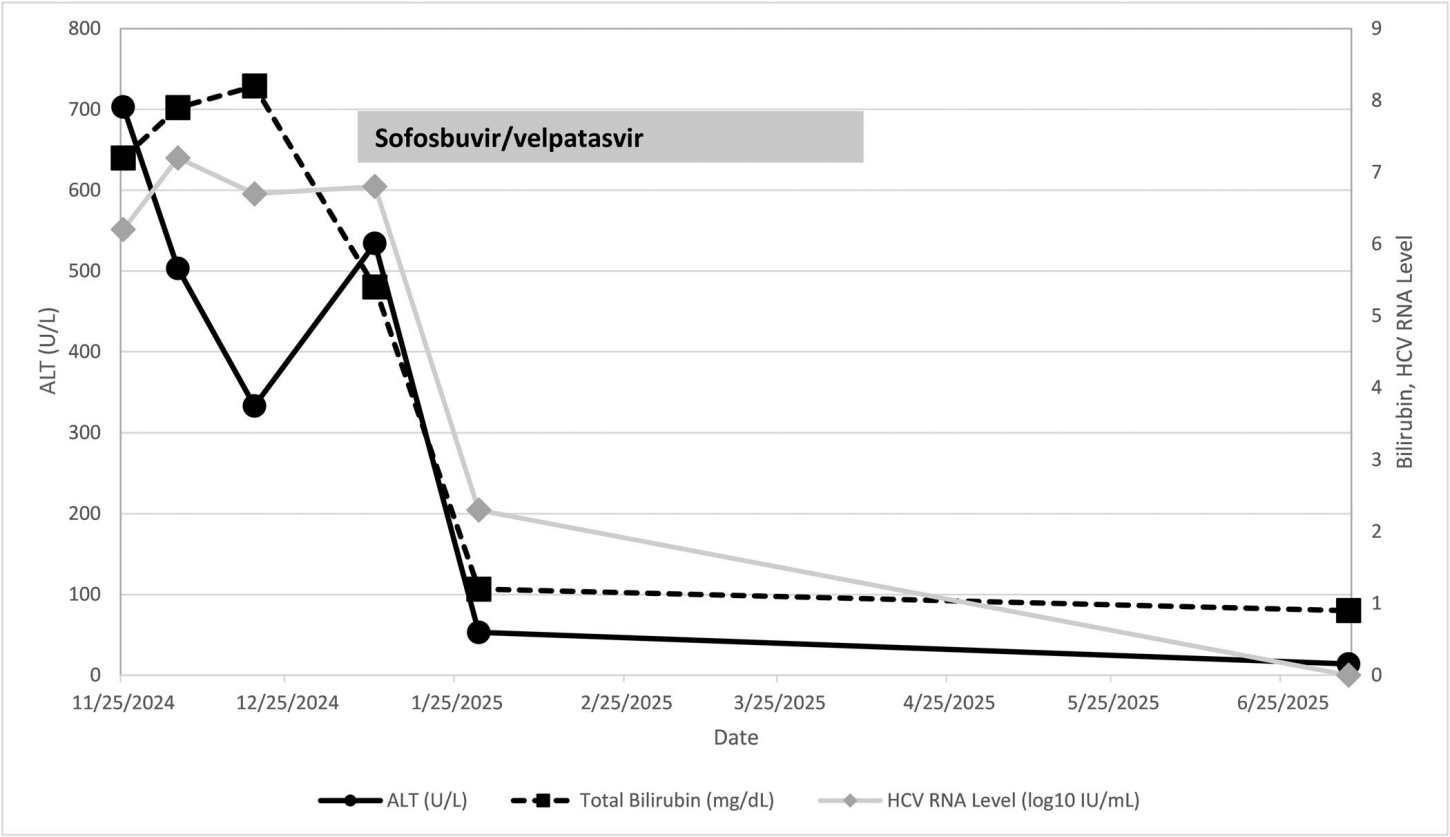
Changes in ALT (U/L), total bilirubin, and HCV RNA from presentation through treatment and post‐treatment follow‐up. ALT, alanine transaminase; HCV, hepatitis C virus.

A formal assessment by our immunology team was negative including normal B‐cell and T‐cell populations by flow cytometry, normal quantitative immunoglobulins, normal CH50, and negative HIV testing. The patient was determined to be immunocompetent on the basis of the clinical and laboratory assessment of our immunology team. We acknowledge that this assessment does not exclude all possible immunologic predisposition to HCV induced liver injury such as subtle defects in HCV‐specific cellular immunity or IL28B/IFNL4 effects.

## 3 DISCUSSION

The pathogenesis of FCH is thought to reflect the direct cytopathic effect of massive viral replication typically compounded by impaired host immunity.^3^ This results in high viral levels, rapidly progressive cholestasis, and histologic findings of hepatocellular ballooning, severe canalicular and intracellular cholestasis with periportal and sinusoidal fibrosis, and minimal inflammatory infiltrate. Unique aspects of HCV‐related FCH include bile ductular reaction and in some cases more significant inflammation with a neutrophil‐predominant interface and periportal infiltrate.^3^

The liver biopsy from our patient did show some histologic features which may be considered discordant with a diagnosis of FCH on the basis of the existing literature, including a mixed inflammatory infiltrate with some eosinophils and the absence of bile ductular reaction. However, expert review at our center and an outside, major pediatric liver center concluded that the biopsy changes were most consistent with FCH. The dramatic response of our patient to treatment for HCV supports HCV as the principal driver of the liver injury.

Prior studies have explored the role of donor and recipient IL28B genotyping in cases of FCH but have been inconclusive.^4^ To our knowledge, the role of hereditary cholestasis syndromes in FCH due to HBV or HCV has not been reported but we found no variants in our patient.^5^ Historically, adults with HCV‐related FCH have suffered very high morbidity and mortality, with the largest experience involving patients affected by FCH after liver transplantation.^6^ However, more recent reports in adults suggest that HCV‐related FCH responds to treatment with direct acting antiviral therapy.^7^, ^8^ Likewise, our patient was treated with sofosbuvir/velpatasvir leading to complete and durable response. We have now followed the patient for 13 months following initiation of treatment.

This case report has important strengths, most notably the availability of longitudinal follow‐up from initial diagnosis through treatment completion and into the post‐therapy period. On the other hand, the maternal history was limited, and we cannot confirm vertical transmission of HCV to the patient. However, given the known maternal history of intravenous drug use and the absence of patient risk factors for acquisition of HCV, vertical transmission was considered to be the most plausible mode of acquisition.

## 4 CONCLUSION

Clinicians should consider FCH in pediatric patients with hepatitis C presenting with acute hepatitis and cholestasis, especially in the setting of high HCV RNA viral level, and consider direct‐acting antiviral therapy when FCH is suspected.

## CONFLICT OF INTEREST STATEMENT

The authors declare no conflicts of interest.

## ETHICS STATEMENT

Written informed consent was obtained from the legal guardian of the patient and written assent was obtained from the patient.

## Supporting information

Supporting File 1.

Supporting File 2.

## Data Availability

The data that support the findings of this study are available on request from the corresponding author. The data are not publicly available due to privacy or ethical restrictions.
