# Supplementary material for: Suspected fibrosing cholestatic hepatitis in an immunocompetent child with hepatitis C: Case report
Source: JPGN Rep. 2026 Oct 15:10.1002/jpr3.70250. Online ahead of print. doi: 10.1002/jpr3.70250 (PMC13670154; doi:10.1002/jpr3.70250)
Supplement: Supplementary file 1 [file JPR3-9999-0-s001.docx]

**Supplementary Material – Patient Perspective**

The following statement was provided to Dr. DiPaola on April 27, 2026 by the patient together with the family.

“We really didn’t know what was happening to [redacted name of patient] before he was seen at the liver clinic at UVA. However, once he was seen, it seemed that we had a good idea about what was wrong and then his treatment fell into place. He has done well since the treatment. He feels entirely well other than some headaches that seem to be related to him needing glasses. We are very grateful for the care that he received.”
