# Supplementary material for: Suspected fibrosing cholestatic hepatitis in an immunocompetent child with hepatitis C: Case report
Source: JPGN Rep. 2026 Oct 15:10.1002/jpr3.70250. Online ahead of print. doi: 10.1002/jpr3.70250 (PMC13670154; doi:10.1002/jpr3.70250)
Supplement: Supplementary file 2 [file JPR3-9999-0-s002.docx]

| ABCB11 | ABCB4 | ABCC2 | ABCG5 | ABCG8 |
| --- | --- | --- | --- | --- |
| ACOX2 | AKR1C4 | AKR1D1 | ALDOB | AMACR |
| ATP8B1 | BAAT | CC2D2A | CFTR | CLDN1 |
| CYP27A1 | CYP7A1 | CYP7B1 | DCDC2 | DGUOK |
| DHCR7 | EHHADH | FAH | GNAS | GPBAR1 |
| HNF1B | HSD17B4 | HSD3B7 | INVS | JAG1 |
| KMT2D | LIPA | MKS1 | MPV17 | MYO5B |
| NOTCH2 | NPC1 | NPC2 | NPHP1 | NPHP3 |
| NPHP4 | NR1H4 | PEX1 | PEX10 | PEX11B |
| PEX12 | PEX13 | PEX14 | PEX16 | PEX19 |
| PEX2 | PEX26 | PEX3 | PEX5 | PEX6 |
| PEX7 | PKD1L1 | PKHD1 | POLG | SCP2 |
| SERPINA1 | SLC10A1 | SLC10A2 | SLC25A13 | SLC27A5 |
| SLC5A1 | SLC5B | SLCO1B3 | SMPD1 | TALDO1 |
| TJP2 | TMEM216 | TRMU | UGT1A1 | UTP4 |
| VIPAS39 | VPS33B |  |  |  |
